# Potential Cost Savings With Low-Dose Abiraterone in the United States

**DOI:** 10.1200/GO.20.00140

**Published:** 2020-05-21

**Authors:** Naveen Premnath, Ramy Sedhom, Arjun Gupta

**Affiliations:** Naveen Premnath, MD, University of Texas Southwestern Medical Center, Dallas, TX; and Ramy Sedhom, MD and Arjun Gupta, MD, Sidney Kimmel Comprehensive Cancer Center, Johns Hopkins University, Baltimore, MD

## TO THE EDITOR:

Patel et al^1^ surveyed more than 100 oncologists in India regarding practice patterns for prescribing low-dose abiraterone. They estimated that, compared with prescribing full-dose abiraterone, routinely prescribing low-dose abiraterone in India would save 182 million USD annually. On an individual patient level, the mean savings would be approximately 2.5 times the per capita income of Indians. We believe that the practice of routinely prescribing low-dose abiraterone should not be restricted to low- and middle-income countries but should be considered across the world, including in the United States.^2^

To estimate the possible savings associated with prescribing low-dose abiraterone in a high-income country like the United States, we analyzed Medicare part D drug spending and utilization data for abiraterone from 2013 to 2017. These data come from a deidentified publicly available database, which includes approximately 70% of Medicare beneficiaries enrolled in part D.^3^ The number of unique Medicare beneficiaries who were prescribed abiraterone increased from 14,188 in 2013 to 19,090 in 2017, as did the cost per 30-day refills, which increased from $6,383 in 2013 to $9,224 in 2017. The aggregate cost paid by Medicare in 2017 was approximately $935 million. Standardizing prescribing to low-dose abiraterone could potentially result in an annual approximate savings of $700 million. Notably, these data do not include commercial payers.

Low-dose abiraterone is listed as an option in the National Comprehensive Cancer Network guideline for treating metastatic castration-resistant prostate cancer (mCRPC) on the basis of a phase II trial that found that low-dose abiraterone (250 mg/day) given with a low-fat breakfast was noninferior to standard-dose abiraterone (1,000 mg/day) while fasting.^4^ This proof-of-concept trial was inspired by preclinical studies on the pharmacokinetics of abiraterone depicting a 5- to 7-fold increase in serum levels of abiraterone with a low-fat diet and an approximately 10- to 17-fold increase when coadministered with a high-fat diet.^5,6^ The primary endpoint of this phase II trial was biochemical—log change in prostate-specific antigen at 12 weeks—not clinical, although the median progression-free survival, decreases in androgen levels, and adverse events of any grade were similar across arms. Importantly, both the prostate-specific antigen response and the time to progression were comparable to previous studies.^7^ Other validated mCRPC endpoints (eg, radiographic progression-free survival and overall survival) have not been studied yet.^8^ This simple strategy is now increasingly relevant, as abiraterone was recently shown to improve survival in the metastatic castrate-sensitive prostate cancer (mCSPC) setting^9^ as well and received US Food and Drug Administration approval for this indication in February 2018.^10^ With expanding use in mCSPC,^9^ a major opportunity exists to lower drug costs by administering the lower dose with food. The National Comprehensive Cancer Network guideline notes the cost savings associated with low-dose abiraterone, which might reduce financial toxicity and improve compliance. The drug is priced at approximately $10,000 per month in the United States for standard treatment but is manufactured and sold in India for approximately one fortieth of this US price (approximately $240 per month).^1^

In summary, low-dose abiraterone represents a strategy that could be associated with notable cost savings, even in high-income countries, such as the United States. High-income countries must learn from initiatives in low- and middle-income countries, just like the reverse; that is the ultimate goal of global collaborations.
